# Burn related injuries: a nationwide analysis of adult inter-facility transfers over a six-year period in the United States

**DOI:** 10.1186/s12873-022-00705-6

**Published:** 2022-08-16

**Authors:** Christopher S. Evans, Kimberly Hart, Wesley H. Self, Sayeh Nikpay, Callie M. Thompson, Michael J. Ward

**Affiliations:** 1 Information Services, ECU Health, Greenville, NC USA; 2 Department of Emergency Medicine, East Carolina University, Greenville, NC USA; 3 Department of Biostatistics, Vanderbilt University Medical Center, Nashville, TN USA; 4 Department of Emergency Medicine, Vanderbilt University Medical Center, Nashville, TN USA; 5 Vanderbilt Institute for Clinical and Translational Research, Vanderbilt University Medical Center, Nashville, TN USA; 6 Division of Health Policy and Management, University of Minnesota School of Public Health, Minneapolis, MN USA; 7 Department of Surgery, University of Utah, Salt Lake City, UT USA; 8 VA Tennessee Valley Healthcare System, 1313 21st Ave South; Oxford House 312, Nashville, TN 37232 USA

**Keywords:** Burns, Inter-facility transfer, Disparities, Emergency medicine

## Abstract

**Background:**

US emergency department (ED) visits for burns and factors associated with inter-facility transfer are unknown and described in this manuscript.

**Methods:**

We conducted a retrospective analysis of burn-related injuries from 2009–2014 using the Nationwide Emergency Department Sample (NEDS), the largest sample of all-payer datasets. We included all ED visits by adults with a burn related ICD-9 code and used a weighted multivariable logistic regression model to predict transfer adjusting for covariates.

**Results:**

Between 2009–2014, 3,047,701 (0.4%) ED visits were for burn related injuries. A total of 108,583 (3.6%) burn visits resulted in inter-facility transfers occurred during the study period, representing approximately 18,097 inter-facility transfers per year. Burns with greater than 10% total body surface area (TBSA) resulted in a 10-fold increase in the probability of transfer, compared to burn visits with less than 10% TBSA burns. In the multivariable model, male sex (adjusted odds ratio [aOR] 2.4, 95% CI 2.3–2.6) was associated with increased odds of transfer. Older adults were more likely to be transferred compared to all other age groups. Odds of transfer were increased for Medicare and self-pay patients (vs. private pay) but there was a significant interaction of sex and payer and the effect of insurance varied by sex.

**Conclusions:**

In a national sample of ED visits, burn visits were more than twice as likely to have an inter-facility transfer compared to the general ED patient population. Substantial sex differences exist in U.S. EDs that impact the location of care for patients with burn injuries and warrants further investigation.

**Supplementary Information:**

The online version contains supplementary material available at 10.1186/s12873-022-00705-6.

## Introduction

Emergency departments (EDs) in the United States (U.S.) care annually for over 400,000 patients with burn related injuries. Approximately 3275 of these result in death [1, 2]. Over the last several decades a network of regionalized burn centers have been developed throughout the U.S. alongside the development of published burn center referral criteria by the American Burn Association [3, 4]. Specialized burn centers are analogous to trauma centers in their availability of institutional resources and burn specialists to care for the significant morbidity and mortality associated with severe burns. Similar to trauma centers, specialized burn centers are limited in their availability and require inter-facility transfer for patients to access a higher level of care.

The ED is an important first step in burn care as up to 92% of burn injuries present to EDs, which are infrequently affiliated with a specialized burn center [5]. In 2008, the U.S. had 128 burn centers (51 ABA verified) [6] across the greater than 4500 hospital-based emergency departments in the country during the same time [7]. Inter-facility transfer of patients with burns, and the appropriateness of these transfers, are important areas of investigation as there is highly variable adherence to burn center transfer criteria, [5, 6, 8–10] contributing to under- and over-triage of patients to burn centers. For example, in a national evaluation of the pediatric population using the Nationwide ED Sample (NEDS), 54% met criteria for appropriateness for referral, while only 8% were actually transferred [11]. In the adult burn population, a contributing factor to potentially inappropriate inter-facility transfers may be the poor provider estimation of Total Body Surface Area (TBSA) [12–14]. However, research on adult burns—which represent a much larger proportion of the population of burn patients—has been under-investigated with most research using single site studies or burn center data registries.

Research on inter-facility transfers of burns and other emergencies have found that non-clinical factors such as insurance status and patient sex are associated with transfers [15–17]. Moreover, inter-facility transfers are events that represent a costly, time-consuming, and disruptive experience for patients and their families, [18] highlighting the need for research in this area. Thus, the primary objective of this study was to describe the patient and hospital characteristics of burn visits to U.S. EDs on a national level and to better understand characteristics associated with inter-facility transfers.

## Methods

This was a retrospective analysis of burn-related injuries presenting to U.S. EDs recorded in the Nationwide Emergency Department Sample (NEDS) from calendar years 2009 to 2014. We limited our timeframe to these years given the switch in diagnostic codes from *International Classification of Diseases, Ninth Revision, Clinical Modification* (ICD-9-CM) to the 10th revision (i.e., ICD-10). NEDS is a nationally representative all-payer dataset of ED visits compiled by the Agency for Healthcare Research and Quality (AHRQ) and includes approximately 20% of all ED visits across the U.S. Using a weighted sampling methodology, NEDS allows for nationally and regionally weighted estimates. A complete description of the methodology behind NEDS sampling and estimates can be found on the AHRQ website [19].

We included all ED visits by adults (age ≥ 18 years) with a burn related ICD-9-CM diagnosis code. A complete listing of included ICD-9-CM codes are listed in the Supplemental Table 1. The primary outcome was inter-facility transfer after initially being evaluated for burn related injuries in the ED and was defined by the variable *EDevent* with “ED visit in which the patient is transferred to another short-term hospital.” Covariates included age, sex, payer, burned body region, visit day, year, hospital region, and hospital type.

We report weighted estimates of burn injury prevalence. Continuous variables were reported as mean and standard deviation or median and interquartile ranges as appropriate, and categorical variables were reported as proportions. Using a multivariable logistic regression model, we estimated adjusted odds ratios and 95% confidence intervals for predictors of inter-facility transfers in burn injured patients. Model covariates were selected *a priori* and included age, sex, payer, interaction of sex and payer, burned body region, visit day, year, and hospital region and type. Only ED encounters with complete case data across covariates were included in logistic regression modeling, and no imputation was performed for missing data. A sensitivity analysis was performed calculating E-Values for covariates in our model, which helps estimate how strong of an effect an unmeasured confounder would need have to question the observed associations between model covariates and likelihood of inter-facility transfer [20]. All statistical analyses were conducted using SPSS 28.0 (IBM Corporation, Armonk, NY) and SPSS Complex Samples Package. This study was exempted by the Vanderbilt University Institutional Review Board.

## Results

**Fig. 1:**
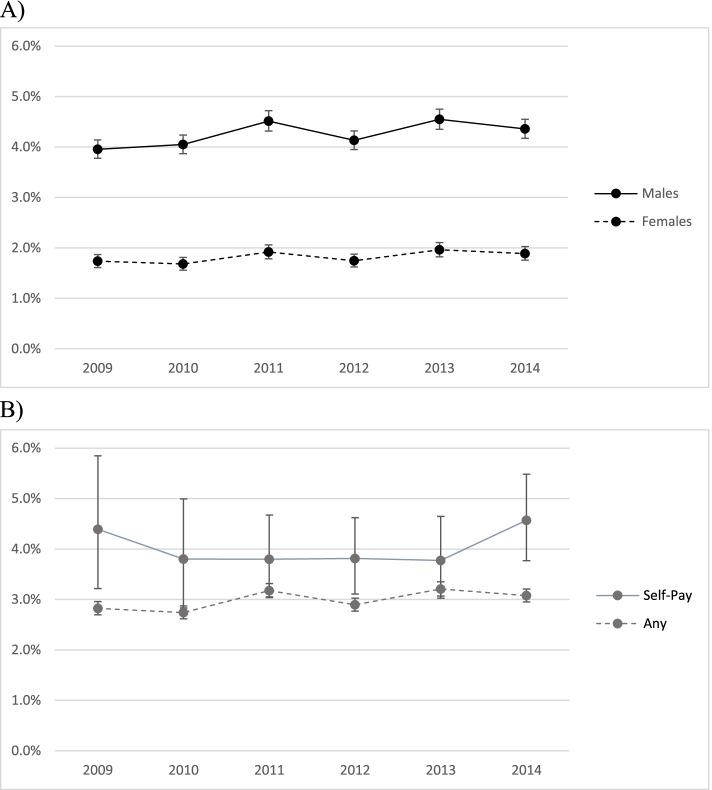
ED visits for Burns with a disposition of Inter-facility transfer during the study period (2009–2014) by **A**) patient sex; and **B**) insurance status (none vs. any)

Among the estimated 794,203,110 overall ED visits during the six-year study period, an estimated 3,047,701 (0.4%) visits were by patients with burn-related injuries in this sample. Among all burn-related ED visits during the six-year period, 108,563 (3.6%) resulted in an inter-facility transfer. Amongst ED visits for any reason in the sample, there were 12,076,331 (1.5%) ED visits that resulted in inter-facility patient transfer. The proportion of burn-related ED visits by sex and insurance status that result in transfer remained steady over the 6-year period (Fig. 1). The mean age among all adult burn patients was 40 (standard deviation 17) years and 47% were female. Among all adult burn patients, private health insurance and self-pay were the most common payment sources (30 and 22%, respectively). Overall, 50% of all adult burn patients had burns involving a high-risk body region (including eyes, face, head, neck, airway, or hands), with 4.2% resulting in inter-facility transfer compared to 2.1% transferred in the burns without involvement of high-risk body regions. Among ED visits with a TBSA recorded of >10, 22.6% resulted in inter-facility transfer, compared to 2.7% among burn visits with TBSA <10%. Additional patient and hospital descriptive statistics are outlined in Table 1, and burn transfers are stratified by high-risk body region as outlined in Table 2.

**Table 1:** Demographic characteristics for ED visits for burn related injuries for the 6-year study period

	Encounter With Transfer (*N* = 108,583)		Encounter Without Transfer (*N* = 2,939,117)		Total (*N* = 3,047,701)
	N	(%)	N	(%)	N
Age (mean, SD)	43	(17)	40	(17)	40 (17)
Age					
18–34	26,307	2.7%	965,205	97.3%	991,512
35–44	12,099	2.9%	404,548	97.1%	416,647
45–54	13,587	3.5%	377,725	96.5%	391,312
55–64	9039	3.8%	227,150	96.2%	236,189
65–74	5486	4.4%	117,965	95.6%	123,451
75–84	2742	4.5%	58,714	95.5%	61,456
>84	1148	4.6%	24,065	95.4%	25,214
Sex					
Male	50,952	4.3%	1,140,348	95.7%	1,191,300
Female	19,426	1.8%	1,034,407	98.2%	1,053,833
Primary Payer					
Medicare	12,835	3.8%	321,646	96.2%	334,481
Medicaid	9998	2.4%	407,500	97.6%	417,497
Self Pay	17,410	3.5%	476,516	96.5%	493,927
Other	9762	3.0%	313,329	97.0%	323,091
Private	20,158	3.0%	649,456	97.0%	669,614
Burns of Eye / Face / Head/ Neck / Airway / Wrists or Hands					
No	23,230	2.1%	1,098,399	97.9%	1,121,629
Yes	47,178	4.2%	1,076,973	95.8%	1,124,152
Region of hospital					
Northeast	7844	1.8%	425,629	98.2%	433,474
Midwest	20,697	3.8%	522,965	96.2%	543,662
South	30,199	3.4%	866,955	96.6%	897,154
West	11,668	3.1%	359,823	96.9%	371,491
Teaching status of hospital					
Metropolitan non-teaching	28,144	3.3%	829,619	96.7%	857,763
Metropolitan	19,890	2.2%	896,794	97.8%	916,684
Non-metropolitan hospital	22,374	4.7%	448,959	95.3%	471,333
Trauma level designation					
Non-Trauma	54,826	3.4%	1,540,201	96.6%	1,595,027
Trauma	14,587	2.4%	584,081	97.6%	598,668
Year					
2009	11,156	3.0%	363,001	97.0%	374,157
2010	11,316	2.9%	375,079	97.1%	386,395
2011	11,898	3.3%	352,311	96.7%	364,209
2012	11,860	3.1%	368,649	96.9%	380,508
2013	11,948	3.3%	349,133	96.7%	361,081
2014	12,231	3.2%	367,199	96.8%	379,430

**Table 2:** Burn-related emergency department encounters, stratified by burn type and region

	Encounter With Transfer (*N* = 108,583)		Encounter Without Transfer (*N* = 2,939,117)		Total (*N* = 3,047,701)
	N	%	N	%	N
940 Burns confined to eye and adnexa	2475	1.6%	152,535	98.4%	155,010
941 Burns of face, head, and neck	29,192	9.5%	279,084	90.5%	308,277
942 Burns of trunk	18,628	6.9%	250,420	93.1%	269,047
943 Burns of upper limb, except wrist and hand	24,887	5.3%	448,624	94.7%	473,511
944 Burns of wrist(s) and hands(s)	27,402	3.7%	716,369	96.3%	743,771
945 Burns of lower limb(s)	19,680	4.2%	454,530	95.8%	474,210
946 Burns of multiple specified sites	3961	17.2%	19,037	82.8%	22,998
947.1 Burn of larynx, trachea, and lungs	373	17.7%	1739	82.3%	2112
947.9 Burn of internal organ unspecified	77	11.8%	574	88.2%	651
TBSA					
948.0, <10%	18,161	2.7%	645,464	97.3%	663,625
948.1, 10–19%	8152	19.1%	34,427	80.9%	42,579
948.2, 20–29%	3612	30.5%	8228	69.5%	11,840
948.3, 30–39%	1909	34.8%	3583	65.2%	5492
948.4, 40–49%	867	31.2%	1915	68.8%	2782
948.5, 50–59%	550	31.6%	1189	68.3%	1740
948.6, 60–69%	301	30.8%	675	69.2%	976
948.7, 70–79%	255	27.8%	663	72.2%	918
948.8, 80–89%	63	11.8%	470	88.2%	533
948.9, >90%	237	6.3%	3500	93.7%	3737
949 Burn, unspecified	997	2.8%	34,388	97.2%	35,385
506 Respiratory conditions due to chemical fumes and vapors	699	1.5%	47,373	98.5%	48,072
692.71 Sunburn	488	0.3%	148,107	99.7%	148,595
692.76 Second Degree sunburn	127	0.4%	34,535	99.6%	34,662
692.77 Third Degree Sunburn	3	0.7%	400	99.0%	404

After controlling for all variables in our multivariable logistic regression model (Table 3), male sex was found to be the strongest predictor of inter-facility transfer (adjusted odds ratio [aOR] 2.4, 95% CI 2.3–2.6). All age groups had decreased odds of transfer compared with the oldest group (>84 years). While the difference in odds decreased with each increase in category there is still considerable overlap in the confidence intervals. Medicare (aOR 1.3, 95% CI 1.2–1.4) and self-pay (aOR 1.3, 95% CI 1.0–1.6) encounters had increased odds of transfer, compared to privately insured. The estimated odds of transfer were lower among those with “other” insurance coverage relative to privately insured. However, there was a significant interaction of sex and payer. The estimated odds ratio of transfer with respect to insurance varied across sex. While those with “other” insurance (which includes worker’s compensation, TRICARE, healthcare for military families, and other state and local government programs) [19] had decreased odds of transfer, males with “other” insurance still had increased odds of transfer (aOR 1.5, 95% CI 1.3–1.7), compared to females with other insurance. Medicare encounters had increased odds of transfer, but males with Medicare had slightly decreased odds of transfer, compared to females. There was no difference in self-pay cases. The remainder of variables can be seen in Table 3. In our sensitivity analysis to estimate potential unmeasured confounding, we found that male sex had an E-Value of 4.24, with an E-Value of the confidence interval closest to the null for male sex of 3.91 (Supplemental Table 2).

**Table 3:** Adjusted odds ratios for inter-facility transfers of patients with burn related injuries between 2009–2014 (*N* = 3,047,701 ED visits)

	Adjusted Odds Ratio (aOR)	95% Confidence Interval	
		Lower	Upper
**Age, (years)**			
18–34	0.5	0.4	0.5
35–44	0.5	0.4	0.6
45–54	0.6	0.5	0.7
55–64	0.7	0.6	0.8
65–74	0.8	0.7	0.9
75–84	0.9	0.8	1.0
>84	ref		
**Sex**			
Male	2.4	2.2	2.6
Female	ref		
**Insurance**			
Medicare	1.3	1.2	1.4
Medicaid	1.0	0.9	1.1
Self	1.3	1.0	1.6
Other	0.7	0.6	0.8
Private	ref		
**High Risk Body Region** (Eye / Face / Head/ Neck / Airway / Wrists or Hands)			
No	0.5	0.5	0.5
Yes	ref		
**Day of Week**			
Weekday	0.9	0.9	1.0
Weekend	ref		
**Region of Hospital**			
Northeast	0.7	0.6	0.7
Midwest	1.3	1.2	1.4
South	1.1	1.1	1.2
West	ref		
**Trauma Center Designation**			
Non-Trauma	1.1	1.0	1.2
Trauma	ref		
**Hospital Teaching Status**			
Metropolitan non-teaching	0.7	0.7	0.8
Metropolitan	0.5	0.4	0.5
Non-metropolitan hospital	1.0		
**Year**			
2009	0.9	0.9	0.9
2010	0.8	0.8	0.9
2011	1.0	0.9	1.0
2012	0.9	0.9	1.0
2013	1.0	0.9	1.0
2014	ref		
Sex * Primary Payer Type Interaction Term			
Male * Medicare	0.7	0.6	0.8
Male * Medicaid	1.0	0.9	1.1
Male * Self Pay	1.2	0.9	1.5
Male * Other	1.5	1.3	1.7
Male * Private	ref		
Female * Medicare	ref		
Female * Medicaid	ref		
Female * Self Pay	ref		
Female * Other	ref		
Female * Private	ref		

## Discussion

In a national sample, this research makes the following two important contributions: First, we provide the first estimates of U.S. adult ED visits inter-facility transfer rates for burn-related injuries. Second, consistent with prior work we found males were more likely to sustain burn related injuries, however we also found marked differences in transfer rates by sex, with males having more than twice the likelihood of being transferred.

Over this six-year study period more than three million patients were evaluated for burn related injuries in U.S. EDs, and 3.6% resulted in an inter-facility transfer, more than double the overall transfer rate of all-comers with non-burn related presentations to the ED. This is consistent with prior research that identified a comparable inter-facility transfer rate amongst North Carolina EDs [5]. Our study findings, which use the largest sample of all-payer datasets in the U.S., reinforces that burn injuries are common and patients with burns present frequently to U.S. EDs. Although inter-facility transfers of ED patients with burn injuries only represent 3.6% of the transfer volume, approximately 18,000 burn-related injuries were transferred annually.

Further, our findings make the important contribution that patient sex and insurance status are independently associated with higher odds of inter-facility transfer of ED visits for burn-related injuries to burn centers. These findings were further supported by E-Values for patient sex and insurance status both greater than 3.0, suggesting a considerable degree of unmeasured cofounding would need to be present to explain away these observed associations. While mortality, length of stay, and rates of surgical operations for burn patients transferred from other facilities and those who initially present to burn centers from scene are similar, [21] the factors associated with inter-facility transfer may be beyond pure clinical factors. In fact, potentially unnecessary inter-facility transfers represent a substantial cost and disruption for patients and the broader healthcare system [22, 23]. However, the appropriateness of transfers in this dataset is not discernible and represents an important future direction.

While variable adherence to the ABA referral criteria has been well documented in both pediatric and adult populations, [9, 11, 24–26] this work identified non-clinical factors independently associated with higher odds of inter-facility transfer. Specifically, we found that male sex and lack of health insurance were the strongest predictors of inter-facility transfer in burn patients. The sex and insurance disparity in inter-facility transfer has been previously described in other clinical conditions. Lack of health insurance is associated with increased inter-facility transfer rates for other time-sensitive emergencies including ST-elevation myocardial infarction (STEMI), [27] as well as across a broad range of medical conditions [28, 29]. For patients presenting to the ED with traumatic injuries, lack of insurance [30] and a patients’ sex [31] have been shown to influence inter-facility transfer decision-making. Treatment at a burn center is also significantly associated with lack of insurance [32]. Further, while severe burns were found to be the single biggest predictor of transfer status for burns, uninsured burn patients were twice as likely to be transferred as those with commercial insurance and this finding did not change with severity of injury [33].

Sex is similarly predictive of transfer. Prior research demonstrates that men disproportionately incur workplace burn injuries [34, 35]. This is similarly seen in national data from the U.S. Bureau of Labor and Statistics. Amongst nonfatal occupational injuries and illnesses, men are more likely than women to incur “days away from work” [36]. This suggests that the nature of the burn and severity, variables not reliably available in NEDS, may impact the transfer decision. However, this finding may be somewhat context dependent; a study in South Africa demonstrated that sex differences in transfers to burn centers only occurred amongst adults. Men were transferred to higher levels of care despite similar severity scores [37]. Thus, while sex differences exist, severity and nature of burn injury alone may not account for transfer differences.

This work contributes to the increasing evidence that non-clinical factors may influence emergency inter-facility transfers. Despite the existence of the Emergency Medical Treatment and Labor Act (EMTALA) of 1986 which was passed by Congress to provide emergency medical treatment for unstable patients presenting to the ED regardless of ability to pay, our findings suggest that non-clinical disparities may also exist in the burn-injured population. One potential explanation is that burn patients with the non-medical factors identified above may disproportionately present to healthcare facilities without burn center capabilities, necessitating transfer. Alternatively, such burn patients may present during busier times of the day when EDs may be crowded and existing resources (e.g., burn service) are overwhelmed necessitating transfer to another facility with the appropriate capabilities. Finally, disparities in inter-facility transfers may exist despite the availability of burn center resources. Future research is needed to better understand why such non-clinical factors may contribute to higher rates of inter-facility transfers for burn patients in emergency care settings.

### Limitations

This study has several limitations. Our findings are based on retrospective administrative data, which uses point estimates from nationally weighted sample of recorded in NEDS. Our study relied on ICD-9 CM diagnosis codes to identify burn related injuries and such codes have their own inherent limitations [38]. Further, our study included data from 2009–2014, after which NEDs datasets started the transition towards ICD-10 Diagnoses codes and only includes three years of complete datasets. Given concerns about diagnoses coding integrity, the availability of data, and to allow comparisons across time, [39, 40] we only included years of NEDs with ICD-9 CM codes. NEDS precluded the identification of transferring facility capability and whether such facilities had burn center resources. Next, based on data availability in NEDs, we were unable to determine the appropriateness of inter-facility transfer as these data are not comprehensive and do not identify if ABA transfer criteria or other clinical characteristics (e.g., need for mechanical ventilation) are present. Furthermore, our findings are derived from burn visits only within the U.S. and may not be generalizable to other countries with different delivery and payment models. Notably, burn size, as measured by total body surface area, was not recorded in two-thirds of ED visits, a key decision variable for burn center transfer. Other notable variables not accounted for that may impact the decision to transfer include depth, concurrent trauma, comorbidities, and carbon monoxide poisoning. Additionally, we were not able to account for other social determinants including housing stability that may influence decision to transfer, nor work-related injuries that may be more common in males. Further, among patients who were transferred to another facility, our data were not able to confirm what proportion were transferred to verified burn centers. Last, this dataset does not provide any information about the pre-existing relationship between transferring and receiving facilities including access to telehealth consultations, both of which may impact both the transfer and location decision.

## Conclusion

In the largest available all-payer national dataset, approximately 18,000, or more than 3 % of all burn-related injuries that presented to U.S. EDs resulted in inter-facility transfers, more than double the national rate. Among burn transfers, male sex and lack of insurance were the strongest independent predictors of increased inter-facility transfer after controlling for patient demographics and hospital characteristics. Further examination of non-clinical factors may facilitate improved understanding and inform interventions aimed at addressing disparities in the emergency management of burn patients.

## Supplementary Information

**Additional file 1: Supplemental Table 1.** ICD-9CM Codes Used to Identify Burn Related Injuries.

**Additional file 2: Supplemental Table 2**. Adjusted odds ratios and E values for inter-facility transfers of patients with burn related injuries between 2009–2014 (*N* = 3,047,701 ED visits).

## Data Availability

The datasets analyzed during the current study are available in the Nationwide Emergency Department Sample (NEDS), collected and maintained by the Agency for Healthcare Research and Quality (AHRQ). https://www.hcup-us.ahrq.gov/nedsoverview.jsp.
