# Supplementary material for: Burn related injuries: a nationwide analysis of adult inter-facility transfers over a six-year period in the United States
Source: BMC Emerg Med. 2022 Aug 16;22:147. doi: 10.1186/s12873-022-00705-6 (PMC9380358; doi:10.1186/s12873-022-00705-6)
Supplement: Supplementary file 1 — Additional file 1: Supplemental Table 1. ICD-9CM Codes Used to Identify Burn Related Injuries. [file 12873_2022_705_MOESM1_ESM.docx]

**Supplemental Table 1.** ICD-9CM Codes Used to Identify Burn Related Injuries

| ICD9 | Burn |
| --- | --- |
| 940 | Burns confined to eye and adnexa |
| 941 | Burns of face, head, and neck |
| 942 | Burns of trunk |
| 943 | Burns of upper limb, except wrist and hand |
| 944 | Burns of wrist(s) and hands(s) |
| 945 | Burns of lower limb(s) |
| 946 | Burns of multiple specified sites |
| 947.1 | Burn of larynx, trachea, and lungs |
| 947.9 | Burn of internal organ unspecified |
|  | TBSA |
| 948.0 | <10% |
| 948.1 | 10-19% |
| 948.2 | 20-29% |
| 948.3 | 30-39% |
| 948.4 | 40-49% |
| 948.5 | 50-59% |
| 948.6 | 60-69% |
| 948.7 | 70-79% |
| 948.8 | 80-89% |
| 948.9 | >90% |
| 949 | Burn, unspecified |
| 506 | Respiratory conditions due to chemical fumes and vapors |
| 692.71 | Sunburn |
| 692.76 | Second Degree sunburn |
| 692.77 | Third Degree Sunburn |
