# Supplementary material for: Burn related injuries: a nationwide analysis of adult inter-facility transfers over a six-year period in the United States
Source: BMC Emerg Med. 2022 Aug 16;22:147. doi: 10.1186/s12873-022-00705-6 (PMC9380358; doi:10.1186/s12873-022-00705-6)
Supplement: Supplementary file 2 — Additional file 2: Supplemental Table 2. Adjusted odds ratios and E values for inter-facility transfers of patients with burn related injuries between 2009–2014 (N = 3,047,701 ED visits). [file 12873_2022_705_MOESM2_ESM.docx]

**Supplemtnatl table 2**. ﻿Adjusted odds ratios and E values for inter-facility transfers of patients with burn related injuries between 2009-2014 (N =3,047,701 ED visits)

|  | **Adjusted Odds Ratio (aOR)** | **95% Confidence Interval** | | **E- Value** | **E-Value for the limit of the CI closest to the null** |
| --- | --- | --- | --- | --- | --- |
|  |  | Lower | Upper |  |  |
| **Age, (years)** |  |  |  |  |  |
| 18-34 | 0.446 | 0.383 | 0.519 | 3.911 | 3.263 |
| 35-44 | 0.488 | 0.418 | 0.569 | 3.515 | 2.911 |
| 45-54 | 0.602 | 0.517 | 0.700 | 2.709 | 2.211 |
| 55-64 | 0.671 | 0.576 | 0.782 | 2.345 | 1.876 |
| 65-74 | 0.786 | 0.675 | 0.915 | 1.861 | 1.412 |
| 75-84 | 0.877 | 0.746 | 1.031 | 1.540 | 1.000 |
| >84 | ref |  |  |  |  |
| **Sex** |  |  |  |  |  |
| Male | 2.404 | 2.241 | 2.580 | 4.241 | 3.909 |
| Female | ref |  |  |  |  |
| **Insurance** |  |  |  |  |  |
| Medicare | 1.253 | 1.135 | 1.382 | 1.816 | 1.526 |
| Medicaid | 0.98 | 0.894 | 1.075 | 1.165 | 1.000 |
| Self | 1.302 | 1.033 | 1.641 | 1.929 | 1.218 |
| Other | 0.683 | 0.598 | 0.781 | 2.288 | 1.880 |
| Private | ref |  |  |  |  |
| **High Risk Body Region**  (Eye / Face / Head/ Neck / Airway / Wrists or Hands) |  |  |  |  |  |
| No | 0.519 | 0.498 | 0.540 | 3.263 | 3.108 |
| Yes | ref |  |  |  |  |
| **Day of Week** |  |  |  |  |  |
| Weekday | 0.907 | 0.87 | 0.946 | 1.439 | 1.303 |
| Weekend | ref |  |  |  |  |
| **Region of Hospital** |  |  |  |  |  |
| Northeast | 0.656 | 0.61 | 0.707 | 2.418 | 2.180 |
| Midwest | 1.304 | 1.224 | 1.389 | 1.934 | 1.748 |
| South | 1.135 | 1.069 | 1.204 | 1.526 | 1.341 |
| West | ref |  |  |  |  |
| **Trauma Center Designation** |  |  |  |  |  |
| Non-Trauma | 1.08 | 1.017 | 1.146 | 1.374 | 1.148 |
| Trauma | ref |  |  |  |  |
| **Hospital Teaching Status** |  |  |  |  |  |
| Metropolitan non-teaching | 0.723 | 0.689 | 0.758 | 2.111 | 1.968 |
| Metropolitan | 0.469 | 0.441 | 0.500 | 3.686 | 3.414 |
| Non-metropolitan hospital | ref |  |  |  |  |
| **Year** |  |  |  |  |  |
| 2009 | 0.852 | 0.796 | 0.912 | 1.625 | 1.422 |
| 2010 | 0.822 | 0.768 | 0.880 | 1.730 | 1.530 |
| 2011 | 0.957 | 0.898 | 1.020 | 1.262 | 1.163 |
| 2012 | 0.933 | 0.873 | 0.996 | 1.349 | 1.068 |
| 2013 | 0.968 | 0.907 | 1.032 | 1.218 | 1.000 |
| 2014 | ref |  |  |  |  |
| **Sex * Primary Payer Type Interaction Term** |  |  |  |  |  |
| Male * Medicare | 0.704 | 0.631 | 0.785 | 2.193 | 1.865 |
| Male * Medicaid | 0.963 | 0.858 | 1.081 | 1.238 | 1.000 |
| Male * Self Pay | 1.184 | 0.911 | 1.538 | 1.651 | 1.000 |
| Male * Other | 1.473 | 1.271 | 1.708 | 2.308 | 1.858 |
| Male * Private | ref |  |  |  |  |
| Female * Medicare | ref |  |  |  |  |
| Female * Medicaid | ref |  |  |  |  |
| Female * Self Pay | ref |  |  |  |  |
| Female * Other | ref |  |  |  |  |
| Female * Private | ref |  |  |  |  |
|  |  |  |  |  |  |
